# Criminological characteristics in women

**DOI:** 10.1192/j.eurpsy.2023.1874

**Published:** 2023-07-19

**Authors:** R. Ouali, N. Smaoui, A. Chamseddine, I. Gassara, R. Feki, M. Maalej, N. Charfi, J. Ben Thabet, L. Zouari, O. Sana, M. Maalej

**Affiliations:** Psychiatry C, Hedi Chaker University Hospital, sfax, Tunisia

## Abstract

**Introduction:**

The female criminal phenomenon will gradually become a reality and the participation of women in criminogenic currents is clearly increasing .Tunisian society, like all other societies, has not escaped this phenomenon.

**Objectives:**

Our objective is to describe the criminological and forensic characteristics of the expertized women.

**Methods:**

This study was retrospective and descriptive. It focused on the files of criminal psychiatric expertise carried out in the psychiatry department "C" at the Hedi Chaker hospital in Sfax and involved female accused subjects.

We have collected all the criminal expert reports carried out over a period of 24 years (from January 1, 1998 to December 31, 2021).

**Results:**

Out of a total of 864 criminal psychiatric expert opinions carried out over a period of 24 years, we collected 56 expert opinions in which the accused was a woman (6.48%).

Among the offenses committed, we identified 31 offenses against persons (55.4%) and 25 offenses against property (44.6%). Homicides represent 37.5% of offenses (N=21) and in second place are thefts (23.2%).

Twenty-seven offenses took place at the victim’s home (48.2%) and 13 (23.2%) at the accused’s home.

The offense was committed under the influence of a psychic disturbance (disorganization, delusional syndrome, psychic excitation, hallucinatory injunctions, cognitive deficit) in 30.4% of cases.

Dementia in the legal sense was retained in 30.4% of cases.

**Conclusions:**

Given the frequency of mental illness among accused women, it would be interesting to optimize their psychiatric care in order to fight against violent acts.

**Disclosure of Interest:**

None Declared

